# An effective sparsity evaluation criterion for power-line interference suppression of EEG signal

**DOI:** 10.3389/fnins.2022.984471

**Published:** 2022-11-25

**Authors:** Zhi-xiang Yang, Bin-qiang Chen

**Affiliations:** School of Aerospace Engineering, Xiamen University, Xiamen, China

**Keywords:** electroencephalogram (EEG), Alzheimer's disease (AD), artificial intelligence, power line interference (PLI), Fourier transform, sparse representation (SR)

## Introduction

Electroencephalogram (EEG) is an electrophysiological signal generated by brain activity in the cranial cavity, which is recorded from the surface of the scalp (Nitish and Tong, 2004). For clinical diagnosis and research, it is an important tool for accurately capturing the electrical activity of the human brain, particularly for monitoring the depth of anesthesia and conducting psychophysiological studies (Ira, 1998; Hosseini and Khalilzadeh, 2010). Furthermore, EEG can be combined with artificial intelligence techniques to identify neurological disorders and syndromes (Paul et al., 2015). Especially for Alzheimer's disease (AD), EEG is a powerful diagnostic tool (Jonkman, 1997; Jacek et al., 2001).

Previously, the early diagnosis of AD was based on the assay of biomarkers, such as amyloid β and phosphorylated protein tau in cerebrospinal fluid. However, cerebrospinal fluid is difficult to access and requires invasive collection procedures. Neuropsychological tests are widely used in the diagnosis of AD as an alternative to physiological tests. But its results are susceptible to multiple factors such as the subjects' educational level. As an enhancement of these methods, new technologies including PET and genetic testing are being used to detect biomarkers or disease-causing genes (Nicolaas et al., 2012). However, these innovative technologies are prohibitively expensive and inconvenient to use, making it difficult for them to gain widespread acceptance. The EEG is a noninvasive, inexpensive, and high-resolution imaging technique that is effective in diagnosing and studying AD. Therefore, EEG has received increasing attention and become one of the most promising methods for early diagnosis of AD.

The EEG observed at the scalp, however, consists of multiple signals. Signals from the cerebral cortex are transmitted to the scalp through volume conductors. During transmission, the EEG signals may be contaminated by external interference, such as baseline wander, EMG interference, and power-line interference (PLI). The cancellation of interference is essential for obtaining more useful information from the actual EEG signal and, therefore, has attracted a lot of attention (Thomas et al., 2001; Michal, 2002; Xinbo et al., 2005). Baseline wander is a low-frequency signal. Commonly used methods to eliminate baseline wander are wavelet transform, median filter, and high pass filter (Lisheng et al., 2006; Mahesh et al., 2008; Antonio and Villani, 2013). EMG interference originates from the contraction and vibration of the internal muscle tissue, which inevitably affects the acquisition of EEG signals. Among the common methods to eliminate EMG interference, ensemble empirical mode decomposition denoising is considered to be more effective (Shing-Hong et al., 2018). Due to effects such as capacitive coupling and magnetic induction, PLI emerges as a major source of interference that leads to the deterioration of signal quality (James and Webster, 1973). To eliminate PLI in EEG, several techniques have been proposed and implemented.

Methods based on the digital notch filter (DNF) to remove PLI are prevalent. Ferdjallah used three different adaptive DNFs to cope with PLI in different cases (Mohammed and Barr, 1994). Nevertheless, there is an overlap between the spectral counterparts of PLI and those of the EEG signal. Due to factors like the Gibbs effect, DNF may introduce severe signal distortion and produce ringing artifacts (Sabine and Dalal, 2019). Discrete wavelet transform (DWT) excels in the separation of signal components and the extraction of transient features. Thenappan utilized DWT to obtain denoised EEG signals (Thenappan, 2021). But DWT performs imperfectly in dealing with a stationary component like PLI. Sparse representation (SR) has received increasing attention in the classification and processing of biomedical signals (Sandeep and Chandra Ray, 2018; Hong et al., 2019; Sunil Kumar and Lee, 2022). It uses linear combinations of atoms from a dictionary to represent a signal. EEG signal classification using the SR-based method has been demonstrated to be highly noise-robust and accurate (Younghak et al., 2015). Gu proposed an SR-based classification model for EEG signal detection to enhance its classification performance (Xiaoqing et al., 2020). Satija proposed a novel sparse representation framework that can adaptively learn dictionaries based on ECG noise types for representing and removing various interference (Udit et al., 2017). The SR-based method is equally effective in suppressing PLI in the EEG. It achieves a better PLI suppression performance in EEG by separating stationary contents from non-stationary contents. However, the sparsity of PLI cannot be achieved when the harmonic information changes abruptly. Hence, to perform this method properly, there is a requirement for pre-checking.

A novel criterion for evaluating the sparsity of PLI is discussed in this article, which is based on the phenomenon of harmonic distortion. The sparsity of PLI will be evaluated by comparing the bandwidth changes of the fast Fourier transform (FFT) spectrum.

## EEG for the diagnosis of AD

Typical pathological features of AD include senile plaque deposition, neuronal fiber tangles, and cholinergic neuron reduction. It has been reported that the reduction of cholinergic neurons is one of the main pathogenesis of AD (Jan Krzysztof and Berse, 2000). As a result, patients with AD experience a rhythmic slowing of the waves on their EEGs. According to their frequency, EEG signals can be divided into five bands: α from 8 to 13 Hz, β from 14 to 30 Hz, θ from 4 to 7 Hz, δ from 0.5 to 3 Hz, and γ above 30 Hz. In general, α and β waves are collectively referred to as fast waves, whereas θ and δ waves are collectively referred to as slow waves. There is an increase in slow waves and a decrease in fast waves in the EEG of AD patients (Yong Tae, 2006). In addition, several studies have reported decreased non-linear structure in the EEG of patients with AD due to a reduction in the dynamic complexity of the brain (Christoph et al., 1994; Jelles et al., 1999). This may be a result of neuronal loss and neocortical disconnection. The EEG can be used as one of the most important tools for diagnosing and classifying AD with so many pathological features shown in it. A classical method of EEG analysis in the clinic is visual inspection, but it is more sensitive to external interference. Following the development of computerized techniques, a variety of mathematical analysis methods are applied to EEG signal analysis, such as wavelet analysis, neural networks, and power spectral density estimation. With these time-frequency analysis methods, the EEG can be further expanded into quantitative electroencephalography (QEEG). The QEEG provides a new perspective for the early diagnosis and study of AD as a complement to visual inspection. Combining computer technology, it is possible to visualize the changes in the cognitive function of patients by displaying the time-frequency characteristics of EEG signals. EEG has increasingly shown its great potential as a noninvasive marker and will play a promising role in the diagnosis and research of AD (Una and Jelic, 2019).

## PLI cancellation methods based on sparse representation

SR-based denoising methods have the advantage of eliminating the problems caused by spectral overlap between PLI and EEG signals. Therefore, the distortion of EEG features during the removal of PLI can be substantially reduced. In conventional signal representation theory, the signal is decomposed on an orthogonal basis. However, conventional methods cannot ensure the sparsity of the results of signal decomposition when analyzing signal components. Sparse representation, as an emerging and reliable signal processing technology, uses linear combinations of atoms from a dictionary to represent a signal. The objective is to obtain an optimal sparse solution of the coefficient vector. However, this requires a proper over-complete dictionary. Thus, the key to achieving sparse representation lies in the selection of the over-complete dictionary.

SR-based denoising theory assumes that the target signal has some fixed features. The target signal component is uniquely correlated to an over-complete dictionary, which ensures its sparsity and prevents other components from being sparsely represented by this dictionary. This is the basis for selecting the over-complete dictionary. According to engineering experience, PLI can be modeled as a sinusoidal component composed of a simple harmonic wave. For determining a sinusoidal wave, only a few harmonic features are required, including amplitude, frequency, and phase. This ensures the sparsity of a sinusoidal signal. Based on the harmonic parameters, the SR-based PLI cancellation method can select the proper over-complete dictionary. Harmonic atoms are well matched to harmonic signals and poorly matched to EEG signal. Therefore, an over-complete dictionary consisting of harmonic atoms can sparsely represent the harmonic signals but not the EEG signal. As a result, the PLI in EEG can be sparsely represented and reconstructed as a compensation signal. PLI suppression is achieved by subtracting the compensation signal from the corrupted EEG signal.

In the literature, the redundant Fourier dictionary, derived from the orthogonal Fourier basis, has been used as an over-complete dictionary (Bin-qiang et al., 2021). Consisting of redundant harmonic atoms with equally spaced frequencies, it can sparsely represent the PLI signal in EEG. After the selection of an over-complete dictionary, the coefficient optimization problem can be solved by matching pursuit (MP) or basis pursuit (BP). Although the current algorithms based on MP or BP have a fast convergence speed, complex iterations are inevitable. The sparse representation can be combined with other spectral analysis methods to reduce computational complexity. Tan developed a dual-step correction algorithm based on implicit sparse representation for suppressing PLI from EEG measurements (Jin-Lin et al., 2021). This does not require a predefined dictionary. The compensation signal will be constructed by estimating harmonic parameters with high precision. Complex iterations are avoided and the efficiency is higher.

The conditions for achieving sparse representation of harmonics can be summarized as single frequency, constant amplitude, and phase continuation. There is an assumption that PLI is a single sinusoidal component in the EEG, but this assumption is often unrealistic. The sparsity of the PLI in EEG cannot be maintained when the harmonic information changes suddenly. Distortion of the PLI signal may thereby invalidate the SR-based PLI cancellation methods. Consequently, it is necessary to establish a criterion for evaluating the sparsity of PLI in advance.

## Criterion for evaluating the sparsity of power-line interference in EEG

Theoretically, PLI is a 50 Hz or 60 Hz (with a variation of ±2 Hz) sinusoidal wave (Kaichen et al., 2022). However, a number of non-linear loads can cause harmonic distortion in the power system (Lundquist, 2001; Muhamad Hafiz Ab et al., 2021). To quantify the level of harmonics, total harmonic distortion (THD) is commonly used as a figure of merit in engineering practice (Muhammad Tanveer et al., 1984). THD is defined as the ratio of the sum of the powers of all harmonic components to the power of the fundamental frequency. To evaluate the sparsity of PLI before applying the SR-based method, however, it is only necessary to determine if distortion has occurred in the PLI without performing precise quantitative measurements such as THD. In the spectrum of a sinusoidal signal, there is only one spectral line and the energy is concentrated within a narrow band. When a change in harmonic information occurs abruptly, however, the sinusoidal signal will inevitably contain high-frequency components. The frequency components other than the fundamental wave will appear in the spectrum of the distorted PLI signal, resulting in a much larger bandwidth. Therefore, harmonic distortion in PLI can be reflected on the basis of this distinct difference.

**Figure 1:**
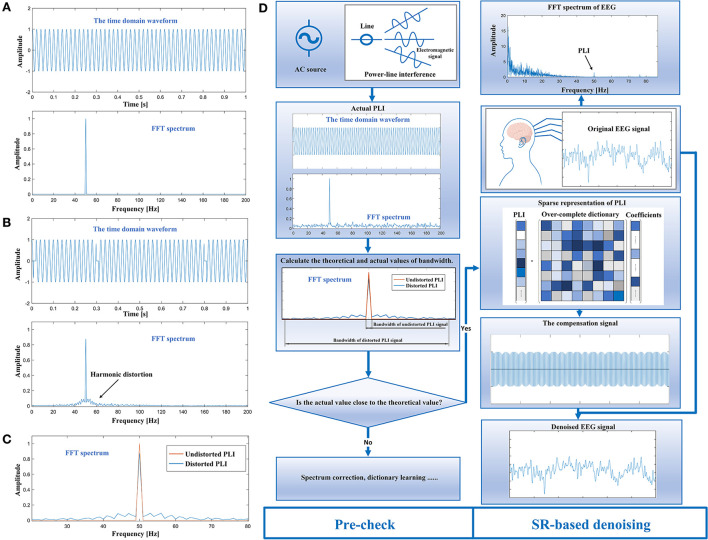
**(A)** Time domain waveform and FFT spectrum of an undistorted PLI signal; **(B)** time domain waveform and FFT spectrum of a distorted PLI signal; **(C)** comparison of FFT spectrum of PLI signals in both cases; and **(D)** the procedure of the proposed method (by Figdraw).

The time domain waveform and FFT spectrum of an undistorted PLI are displayed in Figure 1A. The FFT spectrum illustrates a high concentration of energy within an extremely narrow frequency band area. However, when the PLI distorts, sudden changes in harmonic information can cause harmonic distortion. It is shown that there are some high-frequency components in the FFT spectrum that interfere with components in other frequencies (Figure 1B). Figure 1C compares the FFT spectrum of PLI in both cases. The bandwidth of the FFT spectrum of the distorted PLI signal is significantly larger than that of the undistorted PLI signal. This difference is used in the proposed criterion to determine whether the harmonic information of the actual PLI signal has abruptly changed.

In order to establish the sparsity evaluation criterion, the PLI component is modeled as a single sinusoidal component. After digitization and the FFT, the bandwidth of an undistorted PLI signal can be calculated theoretically. Theoretically, an infinite single sinusoid has zero bandwidth. In practice, a finite sinusoid has a small but non-zero bandwidth. It is a very small theoretical value of about 1 Hz. There are various ways to define and calculate bandwidth. Considering the other frequency components caused by distortion, it is appropriate to use the power bandwidth. Power bandwidth is defined as *f*_2_ − *f*_1_, where *f*_1_< *f* < *f*_2_ defines the frequency band in which 99% of the total power resides (Richard and William, 2003).

PLI is caused by the time-varying electromagnetic fields produced by AC sources and power lines (John, 2009). Hence, it is possible to directly obtain the actual PLI signal for analysis by detecting the power frequency electromagnetic signal in the environment. The proposed criterion requires the calculation of the bandwidth of the FFT spectrum of the actual PLI signal and comparison with its theoretical value. The sparsity of PLI can only be achieved when the actual value is close to the theoretical value. If the actual bandwidth is significantly larger than its theoretical value, additional processing is required, which is shown in the Discussions section.

The steps for implementing the proposed criterion will be presented below. Specifically, the first step requires obtaining the actual PLI signal and performing the FFT to calculate its bandwidth. The second step requires calculating the theoretical bandwidth and comparing it to the actual value. This can be viewed as a simple pattern recognition problem. For example, the ratio of the two values can be used as a discriminant function to determine which pattern class (sparse or dense) the actual PLI signal belongs to. Following the sparsity evaluation, the third step requires a decision about whether to use the SR-based method or to perform additional preprocessing. It is possible to achieve the sparse representation of the actual PLI in EEG after confirming that it is sparse. Thus, the compensation signal can be constructed and subtracted from the original EEG signal to obtain the denoised signal. The procedure of the complete method is shown in Figure 1D.

## Discussions

For harmonic analysis, sparse representation can be a very powerful tool. According to the above arguments, the proposed pre-checking criterion can promote the application of sparse representation in PLI suppression of EEG signals. In the proposed criterion, the difference in the FFT spectrum of the undistorted PLI signal and the distorted PLI signal is utilized. The bandwidth of the distorted PLI signal increases significantly. Therefore, by calculating the actual bandwidth of the FFT spectrum, the sparsity of PLI can be evaluated. It is possible to implement the evaluation criterion algorithmically with the introduction of parameters for quantitative analysis, such as the ratio or difference between actual and theoretical values. In the literature, sample entropy (SampEn) is used as an index for the evaluation of sparsity (Giancarlo et al., 2020). However, SampEN measures suffer from the problem of heavy computations and, hence, are difficult to be used in real-time applications. The proposed criterion based on spectral variation is more efficient for evaluating the sparsity of PLI, which can also be incorporated into other harmonic analysis methods based on SR.

Sinusoidal waves have a limited number of features required to uniquely identify them. Thus, a PLI signal can be easily represented sparsely. In engineering practice, however, it is not always the case that PLI is constant. Once the PLI is confirmed to be distorted, standard dictionaries (e.g., Fourier and wavelet, etc.) cannot capture its precise information. It is therefore necessary to perform some pre-processing before using SR-based methods in such cases. Dictionary learning is an iterative method through which a dictionary can be learned from a collection of signal components (Manas and Das, 2019). In spite of this, dictionary learning requires a significant amount of computation due to the complexity of the matrix multiplication operation. In addition, spectrum correction techniques are used to obtain harmonic parameters using window functions in a more efficient manner (Xie and Kang, 1996; He et al., 2012). As a result, spectrum correction can be used as a means of reducing the distortion of PLI.

## Author contributions

All authors listed have made a substantial, direct, and intellectual contribution to the work and approved it for publication.

## Funding

This work was supported in part by the Natural Science Foundation of China under Grant 62073271, the Fundamental Research Funds for the Central Universities of China under Grant 20720220076, and XMU Training Program of Innovation and Enterpreneurship for Undergraduates (Project 2022Y1486).

## Conflict of interest

The authors declare that the research was conducted in the absence of any commercial or financial relationships that could be construed as a potential conflict of interest.

## Publisher's note

All claims expressed in this article are solely those of the authors and do not necessarily represent those of their affiliated organizations, or those of the publisher, the editors and the reviewers. Any product that may be evaluated in this article, or claim that may be made by its manufacturer, is not guaranteed or endorsed by the publisher.

## References

[B1] Antonio F. Villani V. (2013). Baseline wander removal for bioelectrical signals by quadratic variation reduction. Signal Processing. (2014) 99:48–57. 10.1016/j.sigpro.11, 033.

[B2] Bin-qiang C. Bai-xun Z. Chu-qiao W. Wei-fang S. (2021). Adaptive sparse detector for suppressing powerline component in EEG measurements. Front. Public Health. 473. 10.3389./fpubh.2021.669190 34026718 PMC8137815

[B3] Christoph B. Förstl H. Geiger-Kabisch C. Sattel H. Gasser T. Schreiter-Gasser U. (1994). EEG coherence in Alzheimer disease. Electroencephalogr Clin. Neurophysiol. 90, 242–245. 10.1016/0013-4694(94)90095-7 7511505

[B4] Giancarlo P. Mora-Jimenez I. Jantti R. Caamano A. (2020). Constructing measures of sparsity. IEEE Trans. Biomed. Eng.

[B5] He W. Zhaosheng T. Yong W. Xiaoguang H. (2012). Spectral correction approach based on desirable sidelobe window for harmonic analysis of industrial power system. IEEE Trans Biomed Eng. 60, 1001–1010. 10.1109/TIE.2012.2189531

[B6] Hong P. Cancheng L. Jinlong C. Tao W. Chengjian Z. Xiaoning H. (2019). A novel automatic classification detection for epileptic seizure based on dictionary learning and sparse representation. Neurocomputing. 424, 179–192. 10.1016/j.neucom.12, 010.

[B7] Hosseini S. A. Khalilzadeh M. A. (2010). Emotional Stress Recognition System Using EEG and Psychophysiological Signals: Using New Labelling Process of EEG Signals in Emotional Stress State. In: 2010 International Conference on Biomedical Engineering and Computer Science. p. 1–6. 10.1109./ICBECS.2010.5462520

[B8] Ira J. R. (1998). A. primer for EEG signal processing in anesthesia. J. Am. Anesthesiologists. 89, 980–1002. 10.1097/00000542-199810000-00023 9778016

[B9] Jacek W. K. Gawel M. Pfeffer A. Barcikowska M. (2001). The diagnostic value of EEG in Alzheimer disease: correlation with the severity of mental impairment. J. Clinical Neurophysiol. 18, 570–575. 10.1097/00004691-200111000-00008 11779971

[B10] James H. C. Webster J. G. (1973). 60-Hz interference in electrocardiography. IEEE Trans Biomed Eng. 2, 91–101. 10.1109/TBME.1973.324169 4688314

[B11] Jan Krzysztof B. Berse B. (2000). The cholinergic neuronal phenotype in alzheimer′ s disease. Metabolic Brain Dis. 15, 45–64. 10.1007/BF02680013 10885540

[B12] Jelles B. Van Birgelen J. H. Slaets J. P. J. Hekster R. E. M. Jonkman E. J. Stam C. J. (1999). Decrease of non-linear structure in the EEG of Alzheimer patients compared to healthy controls. Clinical Neurophysiol. 110, 1159–1167. 10.1016/S1388-2457(99)00013-9 10423182

[B13] Jin-Lin T. Zhi-Feng L. Rui Z. You-Qiang D. Guang-Hui L. Min Z.. (2021). Suppressing of power line artifact from electroencephalogram measurements using sparsity in frequency domain. Front Neurosci. 15, 780373. 10.3389/fnins.2021.780373 34776860 PMC8581206

[B14] John G. W. (2009). Medical Instrumentation: Application and Design. John Wiley and Sons.

[B15] Jonkman E. J. (1997). The role of the electroencephalogram in the diagnosis of dementia of the Alzheimer type: an attempt at technology assessment. Neurophysiologie Clinique/Clinical Neurophysiology. 27, 211–219. 10.1016/S0987-7053(97)83777-X 9260162

[B16] Kaichen W. Yang Y. Runwei L. Anyi C. Yaodan X. Lin X. (2022). A capacitive electrocardiography system with dedicated noise-cancellation algorithms for morphological analysis. IEEE Trans Biomed Eng. 61, 1538–1554. 10.1109/TBME.2022.3209325 36155430

[B17] Lisheng X. David Z. Kuanquan W. Naimin L. Xiaoyun W. (2006). Baseline wander correction in pulse waveforms using wavelet-based cascaded adaptive filter. Comput Biol Med. (2007) 37:716–731. 10.1016/j.compbiomed.06, 014. 16930579

[B18] Lundquist J. (2001). On Harmonic Distortion in Power Systems.

[B19] Mahesh C. S. Agarwala R. A. Uplane M. D. (2008). Suppression of baseline wander and power line interference in ECG using digital IIR filter. Circuits, Systems, and Signal Processing. 2, 356–365.

[B20] Manas R. Das S. (2019). Hybrid approach for ECG signal enhancement using dictionary learning-based sparse representation. IET Sci Measurement Technol. 13, 381–391. 10.1049/iet-smt.2018.5060

[B21] Michal T. (2002). Fundamentals of EEG measurement. Meas Sci Rev. 2, 1–11. Available online at: http://www.measurement.sk/2002/S2/Teplan.pdf

[B22] Mohammed F. Barr R. E. (1994). Adaptive digital notch filter design on the unit circle for the removal of powerline noise from biomedical signals. IEEE Trans Biomed Eng. 41, 529–536. 10.1109/10.293240 7927372

[B23] Muhamad Hafiz Ab A. Mokhzaini Azizan M. Sauli Z. Wafiuddin Yahya M. (2021). A review on harmonic mitigation method for non-linear load in electrical power system. AIP Conference Proceedings. AIP Publishing LLC. 10.1063./5.0044251

[B24] Muhammad Tanveer R. Muneeb Afzal M. Muhammad Aaqib S. Ali H. (1984). Analysis and evaluating the effect of harmonic distortion levels in industry. In: 2021 4th International Conference on Energy Conservation and Efficiency (ICECE). IEEE. (2021). 10.1109/ICECE52021, 9406283.

[B25] Nicolaas I. B. Djang D. S. W. Herholz K. Anzai Y. Minoshima S. (2012). Effectiveness and safety of 18F-FDG PET in the evaluation of dementia: a review of the recent literature. J. Nuclear Med. 53, 59–71. 10.2967/jnumed.111.096578 22173840

[B26] Nitish T. V. Tong S. (2004). Advances in quantitative electroencephalogram analysis methods. Annu. Rev. Biomed. Eng. 6, 453–495. 10.1146/annurev.bioeng.5.040202.121601 15255777

[B27] Paul F. Hignett D. Hussain A. Al-Jumeily D. Abdel-Aziz K. (2015). Automatic epileptic seizure detection using scalp EEG and advanced artificial intelligence techniques. BioMed Res Int. 2015. 10.1155./2015/986736 25710040 PMC4325968

[B28] Richard J. C. William S. A. (2003). Telecommunications Breakdown: Concepts of Communication Transmitted via Software-define Radio. p. 138–145.

[B29] Sabine L. Dalal S. S. (2019). Reducing power line noise in EEG and MEG data via spectrum interpolation. Neuroimage. 189, 763–776. 10.1016/j.neuroimage.01, 026. 30639330 PMC6456018

[B30] Sandeep R. Chandra Ray K. (2018). Sparse representation of ECG signals for automated recognition of cardiac arrhythmias. Expert Syst Appl. (2018) 105, 49–64. 10.1016/j.eswa.03, 038.30337072

[B31] Shing-Hong L. Li-Te H. Cheng-Hsiung H. Yung-Fa H. (2018). “Denoising of ECG signal with power line and EMG interference based on ensemble empirical mode decomposition. International Conference on Intelligent Information Hiding and Multimedia Signal Processing”. Cham: Springer. 10.1007./978-3-030-03748-2_21

[B32] Sunil Kumar P. Lee S. W. (2022). Improved sparse representation based robust hybrid feature extraction models with transfer and deep learning for EEG classification. Expert Syst Appl. 198, 116783. 10.1016/j.eswa.2022.116783

[B33] Thenappan S. (2021). Performance improvement in electroencephalogram signal by using DWT. Turk. J. Math (TURCOMAT). 12, 2770–2775. 10.17762/turcomat.v12i10.4895

[B34] Thomas F. C. Luu P. Russell G. S. Tucker D. M. (2001). Scalp electrode impedance, infection risk, and EEG data quality. Clinical Neurophysiol. 112, 536–544. 10.1016/S1388-2457(00)00533-2 11222977

[B35] Udit S. Ramkumar B. Sabarimalai Manikandan M. (2017). Noise-aware dictionary-learning-based sparse representation framework for detection and removal of single and combined noises from ECG signal. Healthcare Technol Lett. 4, 2–12. 10.1049/htl.2016.0077 28529758 PMC5435964

[B36] Una S. Jelic V. (2019). Neurophysiological markers of Alzheimer's disease: quantitative EEG approach. Neurology Therapy. 8, 37–55. 10.1007/s40120-019-00169-0 31833023 PMC6908537

[B37] Xiaoqing G. Zhang C. Ni T. A. (2020). hierarchical discriminative sparse representation classifier for EEG signal detection. IEEE/ACM Trans. Comput. Biol. Bioinform. 18, 1679–1687. 10.1109/TCBB.2020.3006699 32750882

[B38] Xie M. Kang D. (1996). Corrections for frequency, amplitude and phase in a fast Fourier transform of a harmonic signal. Mech Syst Signal Process. 10, 211–221. 10.1006/mssp.1996.0015

[B39] Xinbo Q. Ping Xu Y. Li X. (2005). A CMOS continuous-time low-pass notch filter for EEG systems. Analog. Integr. Circuits Signal Process. 44, 231–238. 10.1007/s10470-005-3007-x

[B40] Yong Tae K. (2006). Quantitative EEG findings in different stages of Alzheimer's disease. J Clinical Neurophysiol. 23:457–462. 10.1097/01.wnp.0000247663, 63. 17016157

[B41] Younghak S. Lee S. Ahn M. Cho H. Chan Jun S. Lee H.-N. (2015). Noise robustness analysis of sparse representation based classification method for non-stationary EEG signal classification. Biomedical Signal Proc Control. 21, 8–18. 10.1016/j.bspc.05, 007.

